# The Functional Properties of Mg–Zn–X Biodegradable Magnesium Alloys

**DOI:** 10.3390/ma13030544

**Published:** 2020-01-23

**Authors:** Dmitry Merson, Alexander Brilevsky, Pavel Myagkikh, Alexandra Tarkova, Alexei Prokhorikhin, Evgeny Kretov, Tatiana Frolova, Alexei Vinogradov

**Affiliations:** 1 Institute of Advanced Technologies, Togliatti State University, 445667 Togliatti, Russia; d.merson@tltsu.ru (D.M.); alexandrbril@yandex.ru (A.B.); feanor357@yandex.ru (P.M.); 2 Centre for Interventional Cardiology, E.N. Meshalkin National Medical Research Centre, 630055 Novosibirsk, Russia; e-artarkova@gmail.com (A.T.); a_prohorihin@meshalkin.ru (A.P.); sibvolna2005@yandex.ru (E.K.); 3 Department of Plant Genetics, Institute of Cytology and Genetics of the Siberian Branch of the Russian Academy of Science, 630090 Novosibirsk, Russia; frolova@bionet.nsc.ru; 4 Department of Natural Science, Novosibirsk State University, 630090 Novosibirsk, Russia; 5 Department of Mechanical and Industrial Engineering, Norwegian University of Science and Technology—NTNU, N-7491 Trondheim, Norway

**Keywords:** bioresorbabale implants, magnesium alloy, deformation processing, microstructure, mechanical properties, corrosion, cytotoxicity

## Abstract

The implantation of metallic devices in orthopaedic surgical procedures and coronary angioplasty is associated with the risk of various adverse events: (i) mechanical (premature failure), (ii) chemo-mechanical (corrosion and corrosion-fatigue degradation) and (iii) biomedical (chronic local inflammatory reactions, tissue necrosis, etc.). In this regard, the development of biodegradable implants/stents, which provide the necessary mechanical support for the healing period of the bone or the vessel wall and then are completely resorbed, has bright prospects. Magnesium alloys are the most suitable candidates for that purpose due to their superior mechanical performance, bioresorbability and biocompatibility. This article presents the results of the comparative research on several wrought biodegradable alloys, assessing their potential for biomedical applications. The Mg–Zn–X alloys with different chemical compositions and microstructures were produced using severe plastic deformation techniques. Functional properties pivotal for biomedical applications—mechanical strength, in vitro corrosion resistance and cytotoxic activity—were included in the focus of the study. Excellent mechanical performance and low cytotoxic effects are documented for all alloys with a notable exception for one of two Mg–Zn–Zr alloys. The in vitro corrosion resistance is, however, below expectations due to critical impurities, and this property has yet to be drastically improved through the cleaner materials fabrication processing before they can be considered for biomedical applications.

## 1. Introduction

Nowadays, a rapid increase in the use of biodegradable and biocompatible materials is seen in medicine [1,2,3]. Considering the success of modern endovascular methods of treatment for coronary heart diseases including congenital heart defects, atherosclerotic lesions of the peripheral arteries, etc., the progress in the development and production of various bioresorbable implants is among the most awaited breakthroughs is the practical area. Modern technologies use various alloys of corrosion-resistant metals, such as nitinol, stainless steel, cobalt-chromium, for the fabrication of stents. However, the permanent presence of a metal scaffold in the human cardiovascular system has serious disadvantages that limit their more extensive use. These include long-term endothelial dysfunction, delayed re-endothelialisation, a higher risk of thrombosis, progressive physical irritation of the vessel wall, chronic local inflammatory reactions, inability to adapt to growth in young patients and the impracticality of the follow up surgical intervention [4,5,6,7,8]. Considering that the remodelling and healing of the stented area occur within 6–12 months, the further presence of the metallic structure is no longer beneficial for the patient [9]. Thus, the development of bioresorbable implants, which provide the necessary support for the healing period of the vascular wall, and are gradually and completely resorbed afterwards without adverse effects for the surrounding tissues, has great prospects for the endovascular surgery. In this regard, magnesium (Mg) alloys have long been recognised as highly potent structural materials for medical applications [10] due to their exceptional combination of high strength, ductility, light weight and low Young’s modulus with natural biocompatibility and biodegradability [11]. However, the degradability plays a dual role in the performance of temporary structures, as uncontrolled degradation may result in premature implant failure or exert an adverse effect on the surrounding tissues due to the excess hydrogen evolution [12,13]. Therefore, modern-day development of temporary metallic implant materials is driven not only by the need for improved mechanical performance, but also by the challenges faced while fulfilling the demanding requirements for biocompatibility and controllable/customisable rate of degradation in a biological environment, yielding corrosive products that have minimal adverse effects on bodily function during the bio-absorption process. Technically, a part of the problem is associated with fundamental limitations on the alloy composition imposed by biocompatibility requirements. In spite of substantial investments in developing biocompatible magnesium alloys over the past two decades, the consensus has yet to be reached on which alloy composition, microstructure and properties are best acceptable for versatile biomedical applications.

A major line of biodegradable alloys aiming at biomedical applications relies upon an Mg–Zn–X archetypal system [14,15,16] (several other interesting systems have been explored too). These alloys have been composed in an array of assortments with different content of Zn up to 6 wt.% and a broad variety of alloying elements X tailored to meet all these multifaceted requirements. Of all compositions, Mg-Zn-Ca (ZX10, ZX40, ZX50) [17,18], Mg–Zn–Zr (ZK60) [19], Mg-Zn-Mn (ZM21) [20], Mg-Y-Zn (WZ21) [21] and their modifications are particularly well-known and noteworthy. The challenges faced by researchers engaged with designing temporary cardio-vascular devices in angioplasty are particularly sophisticated [8]. The small thickness of the struts of the stent stipulates demanding requirements to the alloy’s physical, chemical and mechanical properties [22,23]. For instance, the yield strength should exceed 250 MPa to minimise any possible accidental plastic deformation of the stent during eluting. As plastic deformation cannot be completely avoided during the stent deployment process, the alloy must have considerable ductility and strain hardening ability to avoid strain localisation and premature crack formation. The need to maintain functional properties during the treatment period (about a year) at the heart muscle contraction rate of 70 beats per minute set high requirements for the fatigue resistance at the level of 40 million cycles in a biologically active (corrosive) medium with no loss of integral stability. 

An optimistic perspective for the design of temporary biodegradable vascular stents is brought about by the use of rare earth elements (RE) in the alloy compositions, e.g., see [24] for a practical example of successful development of a RE-containing alloy Mg-2.2Nd-0.1Zn-0.4Zr with a promising combination of properties. The alloying-based design concept relies on the hardening effect due to a multiphase microstructure involving various intermetallic phases. The alloy WE43 developed by Magnesium Elektron Ltd. (UK) with a high content of, Y, Nd and Ga is among the most popular in this category due to a combination of high mechanical properties [25,26] and biocompatibility [27]. In the group of alloys containing RE-elements, the alloys with the so-called Long-range Periodic Staking Ordered (LPSO) structure are of particular interest due to their exceptional combination of functional properties created by the specific chemical and topological ordering in the LPSO phase [28].

Besides the chemical composition, the biomedical properties profile of Mg alloys can be manipulated by tailoring the microstructure by various thermomechanical treatments, of which severe plastic deformation (SPD) is one of the recently emerged tools [29]. What can be stated safely at this stage is that enhancement of mechanical performance (particularly, the ductility and fatigue properties [30,31]) can be achieved with Mg alloys by SPD processes without a loss in their resistance to biocorrosion [32,33,34,35]. Among the procedures devised for deformation processing of wrought Mg alloys, conventional rolling and direct extrusion are the most popular, though they tend to produce the alloys with a strong texture and, therefore, a strongly asymmetrical mechanical response. SPD techniques are of particular interest owing to their capacity to produce significant grain refinement and a broad variety of texture modifications with different strengths [29,36,37,38,39]. The consensus seems to be reached at that there is no universal deformation technique that is “best” suitable for fabricating wrought Mg alloys. Different techniques render different results for different needs and should be used accordingly. For example, the alloys with the LPSO structure benefit strongly from the fibre-like composite structure produced by direct extrusion, whereas many alloys like ZK60, AZ31, Mg-Zn-Ca, etc., exhibit outstanding properties profiles after a combination of various deformation processes such as equal channel angular pressing (ECAP) and extrusion [40,41], or ECAP/extrusion and swaging [31,42], multiaxial forging and rolling [43], etc., [44]. In addition to tremendous hardening, the microstructure refinement often exerts a beneficial effect on the corrosion resistance of many structural materials [45], including Mg alloys [46,47,48]. The improvement in corrosion resistance of multiphase alloys like ZK60 by SPD processing is typically interpreted in terms of grain refinement, and, even more importantly, homogeneity of second phase distribution, and the redistribution of Zn and Zr solutes within the Mg matrix [40,49]. Song and Xu [50] highlighted the significance of the crystallographic texture in the corrosion performance by demonstrating that the basal crystallographic plane showed the higher corrosion resistance of the alloy AZ31 in a chloride-containing solution. Furthermore, concurrent grain refinement via SPD processing and the strong basal texture were claimed as being responsible for the remarkably improved corrosion resistance of nominally the same alloy [51]. On the other hand, the processing route—ECAP or extrusion—was found of little significance for the corrosion rate in this alloy [52]. A comprehensive understanding of the corrosion behaviour in Mg alloys after SPD is, however, still lacking. On the example of the fine grain AZ31 alloy (admittedly, the most widely investigated alloy, which is often used as a benchmark in judging the relative performance of different Mg-based alloys) manufactured by ECAP, it has been demonstrated that smaller grain size increases the materials wettability and bioactivity concurrently with corrosion resistance both in vitro and in vivo [53]. Meanwhile, no any adverse health effects on animals were documented.

The objective of this study was to assess the potential of several typical severely deformed fine grain magnesium alloys and processing techniques to be used for manufacturing biodegradable materials aimed at biomedical applications as bone implants and/or cardiovascular stents.

Based on the experience that some of the present authors accumulated in the area of deformation processing of Mg alloys in the past decade, in the present work, we applied different processing schemes to different alloys to achieve the desired combination of properties. At this stage, we do not intend at comparing the benefits and shortcomings of different technologies for processing of magnesium alloys, which should be the subject of dedicated attention and research aiming at an optimal performance of a specific material.

## 2. Materials and Methods

### 2.1. Alloy Compositions and Processing

Five wrought alloys for the present study were ingot cast from commercial pure components. The chemical compositions of the produced alloys were evaluated by the optical emission spectrometer ARL 4460 (Thermo Fisher Scientific, Waltham, MA, USA) from three to five measurements at different places for each alloy, and the average values for main and some trace elements are listed in Table 1.

The alloy Mg-6Zn-0.5Zr (ZK60) was treated in two notably different ways either using a hybrid processing via direct extrusion followed by two ECAP C-passes through the 120-degree die at 350 °C in a way similar to that described in [40,41] (Specimen #1, denoted in what follows as ZK60(I)) or using multiaxial isothermal forging performed in two subsequent steps at 400 °C and 300 °C, respectively, as described in [43,54] (Specimen #2, ZK60(II)). Alloys Mg-4Zn (#3), Mg-1Zn-0.1Ca (#4, ZX10) and LPSO Mg-1Zn-2.9Y (#5, WZ31) were processed by ECAP at different temperatures to ensure their deformability without cracking, and the LPSO alloy Mg-2Zn-5.7Y (#6, WZ62) was extruded at 350 °C. Equivalent imposed strains for all processing routes are shown in Table 2.

### 2.2. Microstructure Characterisation

For metallographic observations, the sections of the alloys were mechanically polished using the sandpaper, diamond suspensions and colloidal silica to the 0.25 μm grade. Finally, they were etched in a solution containing 50 mL distilled water, 150 mL ethanol, and 1 mL acetic acid for 10 to 30 s at room temperature. Microstructural observations were carried out using an optical microscope Zeiss Axiovert (Carl Zeiss, Ulm, Germany) with the Thixomet image processing software (Thixomet, St. Petersburg, Russia) and a field emission gun scanning electron microscope (SEM) Zeiss SIGMA (Carl Zeiss, Ulm, Germany) equipped with the EDAX/TSL (EDAX, Mahwah, NJ, USA) electron backscattered diffraction (EBSD) detector.

### 2.3. Mechanical Testing

The mechanical properties were assessed in monotonic tensile tests performed using a rigid screw-driven testing machine by Kammrath & Weiss (Kammrath & Weiss, Dortmund, Germany) at the nominal strain rate of 1 × 10^−4^ s^−1^ at room temperature in air. The dog bone specimens with 10 mm gauge length and 3 × 4 mm^2^ cross section were shaped by spark erosion in the longitudinal direction of the worked billets. All specimens were mechanically polished to a mirror finish prior to testing.

### 2.4. Corrosion Testing

The corrosion rate measurements were performed by two independent methods—hydrogen evolution [55] and weight loss [56]—on series of three samples of each alloy immersed into a simulated body fluid (SBF) for 168 h in the thermally stabilised at 37 ± 1 °C bath with of 3 l SBF. The Ringers’ SBF containing 8.6—NaCl, 0.3 g KCl, 0.3 g CaCl_2_ and 0.25 CaCl_2_ · 6H2O in 1 L of distilled water was used. During the test, the pH value was measured by the I160 digital pH-meter (Mettler Toledo, Columbus, OH, USA) and automatically corrected to be at 7.4 ± 0.4. The pH correction solution contained 500 mL of Ringer’s solution and 0.5–1 mL of phosphoric acid.

The specimens of 7 × 7 × 2 mm (#1, #2, #3, #5) and 5 × 10 × 2 mm (#4, #6) dimensions were ground by sandpaper down to the #2500 grade, washed in an ultrasonic bath in acetone and quickly fan-dried and weighted on the analytical balance with the accuracy of 0.0001 g. After testing, the samples were again washed in acetone, dried and weighed. Then corrosion products were removed by rinsing in a solution consisting of 200 g CrO_3_, 10 g AgNO_3_ and 1000 mL distilled water. After removing the corrosion products, the specimens were washed several times in ethanol, dried and weighted again. All tests were performed in a room with controlled temperature and relative humidity (26.0 ± 3.0 °C and 65 ± 15%, respectively).

### 2.5. In Vitro Toxicity Testing

The in vitro cytotoxicity tests were performed using several methods.

To evaluate the cell viability, the MTT assay method [57] was used according to the ISO 10993-5:2009 [58] standard. The extracts were prepared using the immortalized human fibroblasts (American Type Culture Collection—ATCC (ATCC, Manassas, USA)—number CRL-4058). One-hundred microliters of cell suspension (3 × 10^4^ cells/mL density) was dispensed into a well of 96-well plate and incubated for 24 h. Then, the medium was replaced with the prepared extracts from the alloys. To account for the concentration dependence of the extract, dilutions of 1:2, 1:4, 1:6, 1:8 and 1:10 were prepared. The experiment was repeated three times for each alloy sample. The culture medium was used as a negative control, and doxorubicin, a cytotoxic agent, was used as a positive control. After 24 h of incubation, 3-(4.5-dimethylthiazol-2-yl)-2.5-diphenyl-tetrazolium bromide (Sigma, St. Louis, MO, USA) was added in the amount of 10 μL per well (concentration of the dye was 3 mg/mL in PBS). After 2 h of incubation, the medium was removed. The resulting formazan was dissolved in 100 μL of isopropanol per well (Sigma) and the absorbance measurement was performed using a MultiScan FC flatbed spectrophotometer (Thermo Fischer Scientific, Waltham, MA, USA) at the wavelength of 620 nm. The result is presented as the average cell survival (in%) based on the results of 3 measurements ± SEM.

Microscopic analysis was performed on intravital cell samples. For this, the cells were planted in the wells of a 6-well plate (10^4^ cells per well) and incubated for 24 h to attach. Then, the medium was replaced with an extract from alloys (2 mL per well). The cells were incubated for 24 h. Then, the microscopic analysis weighted using an inverted microscope AxioVert Observer Z1 (Carl Zeiss, Ulm, Germany). The images were obtained and processed using the Zen 2 (Carl Zeiss, Ulm, Germany) software. The culture medium was used as a negative control, and mitomycin cytostatic was used as a positive control.

Flow cytofluorometry was performed with an Attune NxT (Thermo Fischer Scientific, Waltham, MA, USA) flow cytometer. After the microscopic analysis, the cells from the wells were removed using trypsin and washed twice with 1 mL PBS. After the second wash, the cells were suspended in 1 mL of PBS, stained with propidium iodide (Thermo Fischer Scientific, Waltham, MA, USA) and then analysed using a cytofluorimeter. The final result is presented in the form of the ratio of normal and dead cells in the analysis of at least 30,000 cells. The pH value of the culture medium was measured using a SevenCompact pH/Ion S220 pH-meter (Mettler Toledo, Columbus, OH, USA) with an accuracy of ±0.01 pH.

## 3. Results

### 3.1. Microstructure

**Figure 1:**
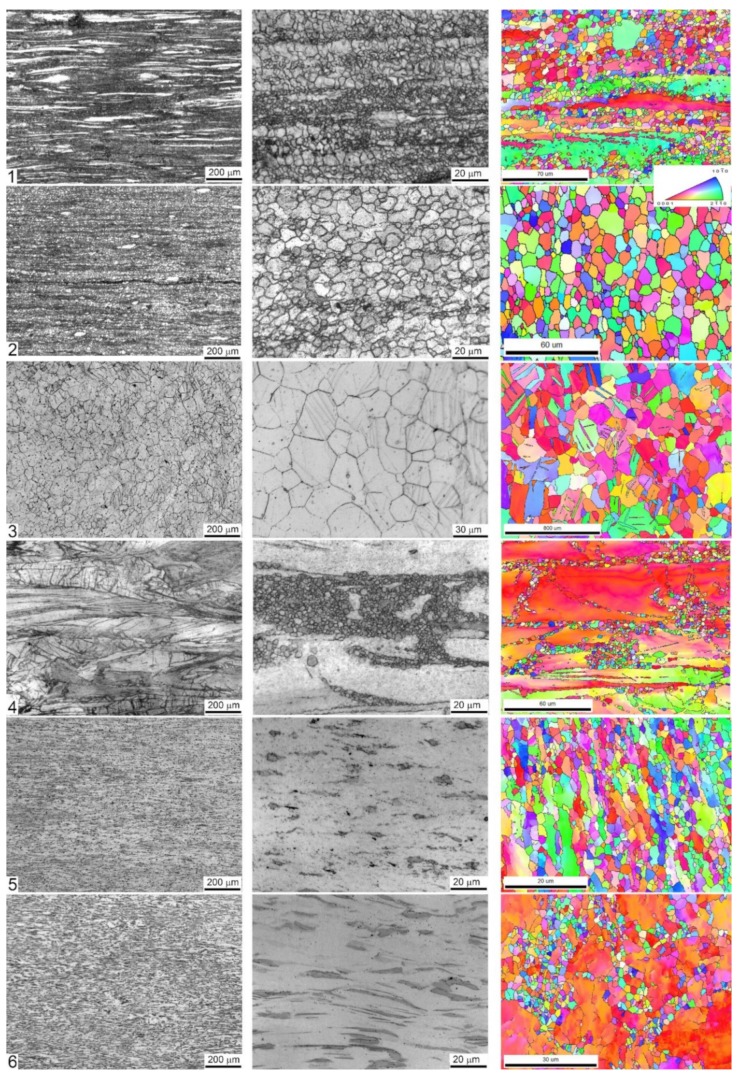
Microstructures of longitudinal sections of six specimens tested. Each raw corresponds to one specimen labelled 1 to 6 according to the specimen number as in Table 1, Table 2, Table 3 and Table 4. Optical microscopy images taken at different magnifications are shown in greyscale; the electron backscattered diffraction (EBSD) images are displayed in the inverse pole figure colours shown in the inset.

Figure 1 represents the optical and SEM EBSD microscopy images characterising the typical fragments of the microstructure of the alloys. The observations reveal that the microstructures of the examined specimens are notably different in terms of grain dimensions and morphology resulting from different processing schemes. The differences in the microstructures were expected in view or remarkably different phase compositions of the alloys studied. Not only the phase composition affects virtually all physical and chemical properties of a metallic alloy, but it strongly influences all aspects of microstructural changes that occur during strain-dependent plastic deformation under elevated temperature. The processing schedules used in the present work involve significant plastic deformation at high homologous temperatures when dynamic recrystallisation (DRX) dominates. Moreover, it is DRX that governs the microstructural evolution towards the fine equiaxial grain structure through the grain nucleation and growth processes in Mg alloys [59,60,61]. The nearly completely recrystallised uniform microstructure is obtained in the specimens #2 (ZK60 alloy after MIF processing), #3 (Z4 Mg-4Zn), and #5 (WZ31 Mg-1Zn-2.9Y alloy after ECAP at 425 °C). For other alloys, the DRX is obviously incomplete, and a large volume fraction of deformed and elongated initial coarse grains coexist with fine recrystallised grains of 1–5 μm size, giving rise to a bimodal grain distribution.

The fine DRX grains nucleate primarily at stress risers close to the initial grain boundaries as is particularly clearly seen for Specimen #4 (extruded ZX10). The DRX process completes when the DRX grains replace all the initial grains, and the average grain size reaches a steady value. Even though the DRX itself results in a very fine grain microstructure, the DRX grains can eventually coarsen due to rapid grain growth at high processing temperatures. This can be seen, for example, for Specimen #3, Mg4Zn, showing the uniform postprocessing grain structure with the average grain size of 126 μm with the large standard deviation of ± 86 μm and multiple deformation-induced twins, which could not be avoided during ECAP even at the significantly elevated temperature of 415 °C. Area average grain sizes (edge grains and twin boundaries exclusive) are shown in Table 2.

### 3.2. Mechanical Properties

**Figure 2:**
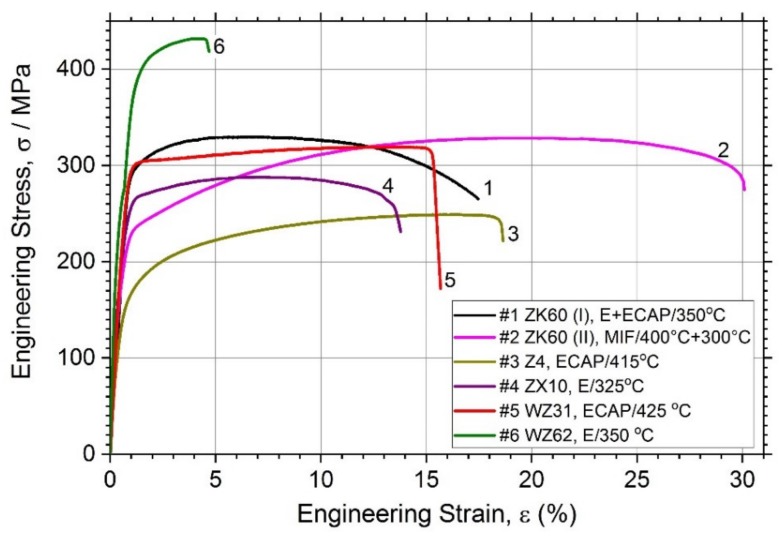
Tensile stress–strain curves for different magnesium alloys studied.

Figure 2 showing the tensile stress-stress diagrams and Table 2 together summarise the mechanical properties of the alloys studied.

In line with common expectations, the simple binary Z4 Mg-4Zn alloy (Specimen #3) exhibits relatively low tensile strength (lowest among others)—the tensile yield stress σ_0.2_ and the ultimate tensile strength σ_UTS_ are of 126 MPa and 250 MPa and, respectively, whereas the ductility (elongation at break, *ε*_f_) in excess of 18% is quite good, which complies with the minimum expectations for the alloys aiming at biomedical applications [12]. Compared to these values, significant strengthening is achieved in all other tertiary alloys, of which the highest tensile strength properties (σ_0.2_ = 356 MPa, σ_UTS_ = 430 MPa) are obtained for the alloy WZ62 due to the integrative effect of the uniformly distributed LPSO phase and finer grain size of the α-Mg matrix. However, the elongation at break in this alloy is compromised if compared to others. On the other hand, the popular alloy ZK60 exhibits an excellent combination of strength and ductility: the strength comparable (or even higher) to that in the conventionally extruded bars in the peak-aged T6 or T5 condition [19,62] and the ductility in excess of 16% for the alloy (I) and of 30% for the alloy (II), depending on the processing route. In combination with the σ_UTS_ value as high as 330 MPa, this is a very remarkable result. For the sake of comparison, Chen et al. [19] reported the *ε*_f_ values for the alloy ZK60 in the ex-extruded, T5 and T6 conditions at 19.6, 17.9, and 23.2, respectively (similar results were also obtained in [63] for rolled ZK60). It seems plausible to believe that the superior combination of mechanical properties observed in the alloy ZK60 (II) (Specimen #2) is due to a combined effect of grain refinement down to a micrometre scale and texture modification caused by two-step multiaxial forging; this dual effect of SPD has been well established and often claimed as advantageous in gaining extra ductility in Mg-based alloys [64,65]. Overall, all alloys in the present study exhibit the mechanical properties profiles suitable for stringent biomedical applications: the range of mechanical properties demonstrated by different alloys is broad and tailorable, which agrees with the variability of microstructures obtained in the course of processing.

### 3.3. Corrosion Resistance

**Figure 3:**
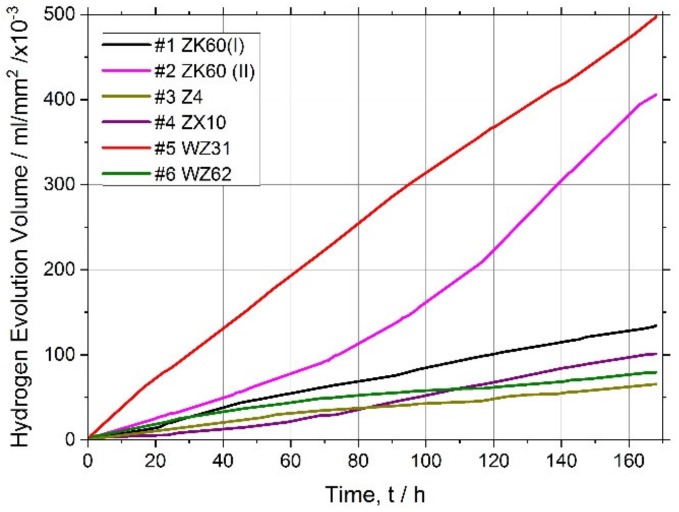
Hydrogen evolution curves for the magnesium alloys tested.

**Figure 4:**
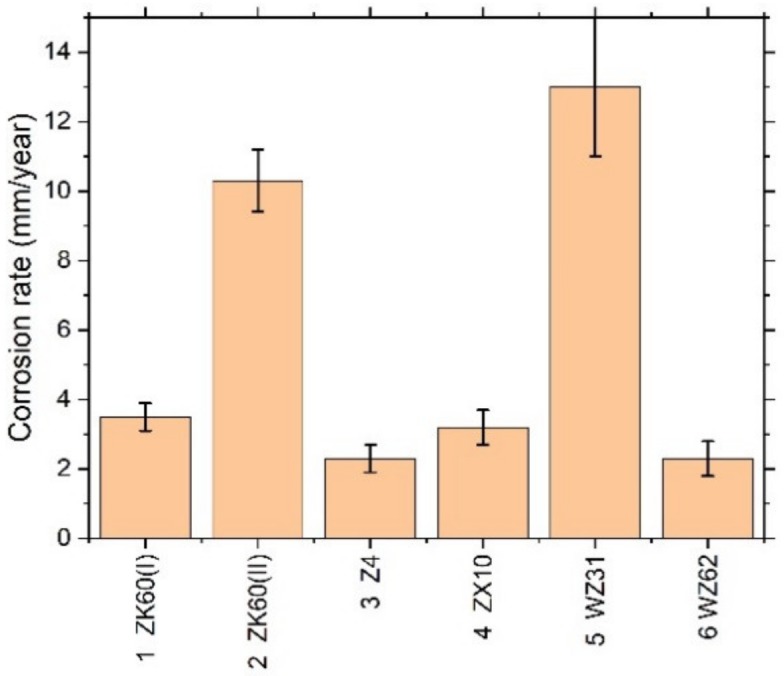
Average corrosion rate assessed by the hydrogen evolution method for different Mg–Zn–X alloys immersed in the SBF for 168 h.

It has long been recognised and generally accepted that the corrosion rate of magnesium and its alloys in aqueous chloride solutions can be quantitatively represented by the amount of hydrogen gas evolved (volumetrically) in the reaction [55,66,67]. Typical hydrogen evolution curves under pH-controlled conditions are shown in Figure 3 for all alloys studied. Values of the average corrosion rate for different Mg alloys immersed in the SBF for 168 h are summarised in Table 2 and Figure 4 (averaged over three specimens).

Kirkland et al. [68], in their comprehensive survey, highlighted that the corrosion rate of Mg alloys can vary over a range of three orders of magnitude. In the present work, the corrosion rates also ranged widely from of 13 mm/year for the alloy WZ31 to of 2.3 mm/year for Z4 and WZ62, thus falling in a category of most popular magnesium alloys showing similar performance in 7-days SBF tests [68,69]. For comparison, high purity Mg exhibits typically of 0.9 mm/year SBF corrosion rate [70]. Admittedly, the biodegradation rate observed in the present work is not exceptional in either positive or negative sense compared to many Mg-based alloys, and there is still a lot to desire in the biocorrosion rate, which is supposed to be less than 0.5 mm/year [12]. Furthermore, it can be remarkably better than this targeted value. For example, an excellent biocorrosion performance has been achieved in the very high-purity WZ21 alloy [71] (0.35–0.55 mm/year measured by the weight loss method on the basis of 7 days immersion into Nor’s solution).

Figure 1 evidently shows that neither of the alloys tested can be regarded as microstructurally homogeneous. Even though the grain distribution is reasonably uniform in the specimens #2 ZK60(II) and #5 WZ31, the second phases are abundantly present and inhomogeneously distributed. The second phases usually act as cathodic sites significantly accelerating the corrosion processes in magnesium alloys by a micro-galvanic effect (with notable exceptions for some Ca phases [17]). The decrease in size of second phase particles and the resulting increase in the microstructural homogeneity usually decrease the localisation of corrosion.

**Figure 5:**
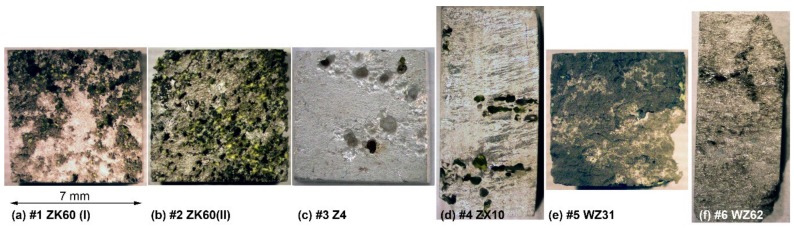
Optical macro-morphologies of the Mg alloys specimens after immersing in Ringer SBF for 168 h and corrosion product removal: (**a**) ZK60(I), (**b**) ZK60(II), (**c**) Z4, (**d**)ZX10, (**e**) ZX10 and (**f**) WZ62.

Both ZK60 specimens exhibited particularly severe heterogeneous corrosion patterns over the whole surface Figure 5a,b, that correlates with the structural (and compositional) inhomogeneities visible in Figure 1. Of these two alloys, Specimen #1 showed a less damaged surface with some parts only lightly touched by corrosion, while Specimen #2 was severely corroded over the whole surface. Thus, the corrosion rate correlated with the corrosion morphology for these specimens. The binary Z4 Mg-4Zn specimen #3 and tertiary ZX10 Mg-0.9Zn-0.2Ca alloys #4 showed very heterogeneous damage profiles with deep corrosion cavities on the surface. Elsewhere, the surfaces were just lightly affected by the aggressive environment. The Y-containing alloys (#5 and #6) exhibited notably more uniform corrosion over the whole specimen surface when compared to strongly localised (pitting) degradation of Mg–Zn alloys with Ca or Zr (#1–#4), Figure 5. Of these LPSO alloys, Specimen #6 had the notably less deep corrosion damage relief, again correlating with the lower corrosion rate.

It is hypothesised that the main reason for the lower than desired corrosion performance is the low purity of the cast alloys, which needs to be improved (particularly for such trace elements as Fe, Cu, Ni to be lower than the tolerance limits [70]). When the impurities content exceeds the tolerance limits, corrosion progresses rapidly and continues at a high rate. The present investigation corroborates this common trend. The alloy WZ31 (specimen #5) has the concentration of Fe exceeding the tolerance limit of 180 ppm, giving rise to the high degradation rate and poor corrosion resistance. Although the concentrations of other trace elements are notably lower than the tolerance limits, there still seems to be room to improve the purity of the alloys. All in all, the corrosion resistance of Mg alloys in chloride solutions in general and in the physiological environment, in particular, is determined by multiple factors, including the composition, impurities, secondary phases, and, to a lesser extent, a grain microstructure and texture resulting from the manufacturing process [11,72].

### 3.4. Cytotoxicity Test

Results of the MTT test are presented in Table 3 as the average cell survival percentage of three independent measurements ± SEM for each condition.

The concentrated extract from Specimen #2 exhibited pronounced cytotoxicity. However, upon dilution by the medium, the adverse effect of the medium on the cells is alleviated. A weak cytotoxic effect can be noticed in Specimen #5 in the concentrated solution.

**Figure 6:**
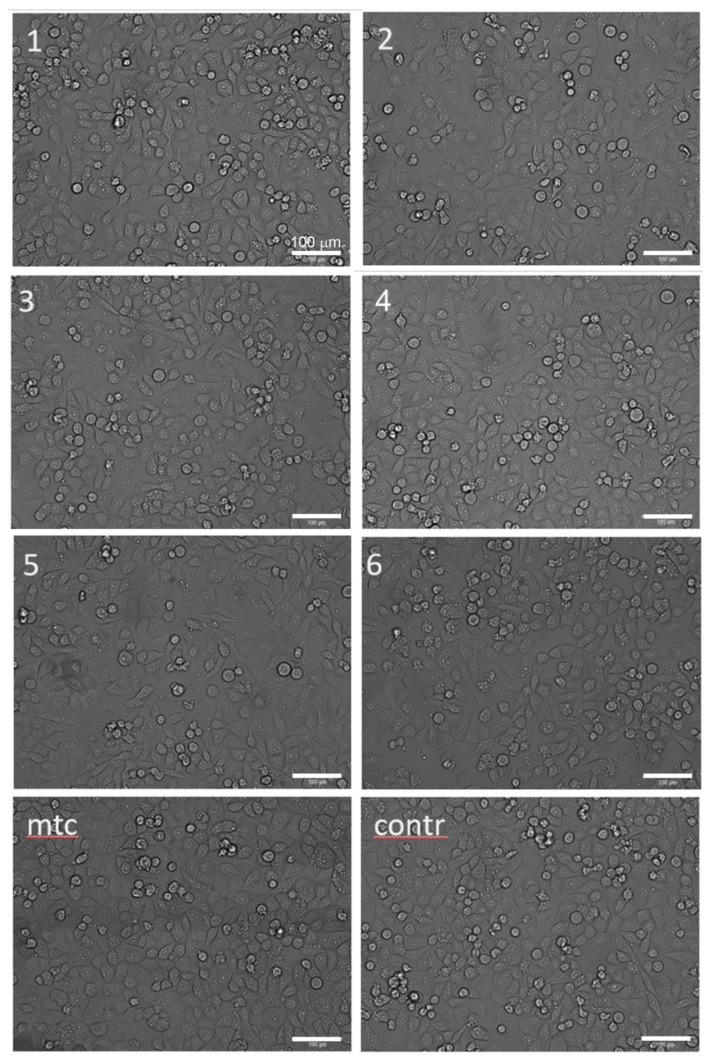
Optical morphologies of CRL-4058 cells after 24 h incubation with extracts of alloys (micrographs are labelled according to the specimen numbers 1–6 as in Table 1, Table 2, Table 3 and Table 4); here, *mtc* stands for the positive control (cytostatic mitomycin) and *contr* stands for the negative control (cells with culture medium); the scale is the same for all micrographs.

The microscopic analysis was performed to elucidate the changes in the cell morphology occurring after 24 h of incubation with extracts. The result is presented in Figure 6. It shows that the morphology of cells did not change appreciably when exposed to extracts. The visually noticeable lower density of cells in the field of view for cytostatic mitomycin micrographs for Specimens #2 and #5.

**Figure 7:**
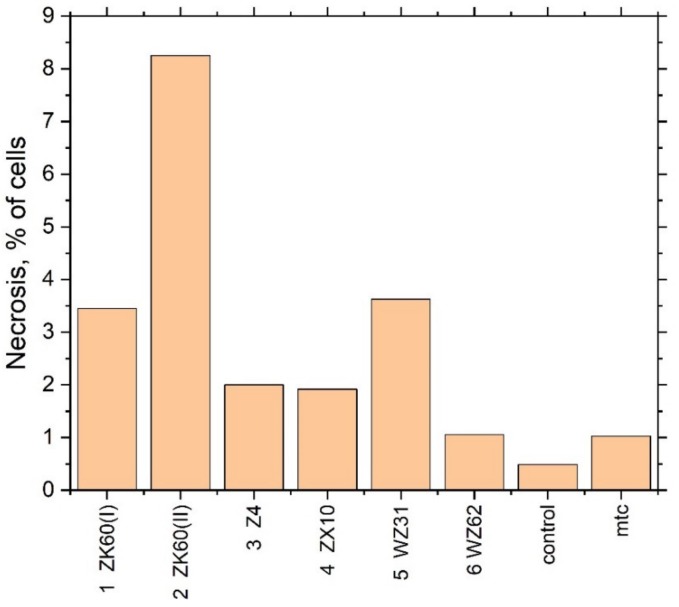
The fraction of necrotic cells in the extracts of different Mg alloys.

To clarify the mechanism of the toxicity of the extracts (cytostatic or cytotoxic), a cytofluorometric analysis of the cells stained with propidium iodide was performed. In the case of a cytostatic mechanism, the proportion of dead cells in the population should not increase, whereas, in the case of intensified necrosis, a significant increase in the percentage of dead cells (stained with propidium iodide) is expected. Results are presented as a proportion of necrotic cells in the population of at least 60,000 cells (Table 4 and Figure 7).

A significant increase in the fraction of necrotic cells was observed when the cell culture was incubated with the extract of Specimen #2 in good agreement with the results of the cytotoxicity test. Summing up the mutually supporting findings from the MTT assay test and flow cytofluorometry, it can be concluded that the toxicity of Specimens #2 and #5 is due to cell death. Interestingly, Specimen #1 (ZK60 (I)), having nearly the same chemical composition as Specimen #2 (ZK60 (II)), did not show significant cytotoxicity in the MTT test, although the trend for an adverse effect can be noticed.

As the alloys contain magnesium, which can form a hydroxide when reacting with water and alkalise the medium with the adverse effect on living cells, the pH of the extraction medium was measured after incubation of the extracts with the cells. Results are presented in Table 4. It is seen that the pH value of the alloys extracts is higher than that of the control. Corroborating the previous findings, the highest pH value is observed for Specimens #2 (ZK60 (II)) and #5 (WZ31) which exhibit the most appreciable cytotoxic effect.

## 4. Discussion

The combination of mechanical properties of most of the alloys tested, except for Z4, appeared to be superior to those usually reported in the alloy AZ31 manufactured by conventional or SPD processing routes [44,73,74]. The same picture emerges if one compares the alloys with similar chemical compositions: c.f. Mg-4Zn [16], ZX10 [75] or ZX20 [76], and ZK60 alloys [41] (see also a review [65]).

All results of biomedical tests converge in that despite the vast difference in the microstructures produced by different processing techniques in different groups Mg-based alloys, there is only one potentially toxic alloy—the fine-grain #2 ZK60 fabricated by multiaxial isothermal forging. The mild toxicity effect in this alloy is due to the increase in the fraction of necrotic cells in the concentrated extract. It was shown that incubation of cells with extracts of alloys does not change their morphology. Besides, all samples incubated with cell cultures increase the pH of the medium. However, for the #2 ZK60 (II) alloy, with the most pronounced toxic effect, the highest pH value exceeding the physiological norm was observed, which may explain the increased cell necrosis after incubation with the extract. As it has been noticed above, of two differently produced alloys ZK60 and with nearly the same chemical composition only one (#2) showed the unacceptable toxicity at the largest concentration of the extract while the other (#1) did not. It is, therefore, plausible to conclude that the toxic effect is not related to the nominal chemical composition of the alloy. Neither had we found any trace elements among those detected by the optical-emission spectral analysis, which could possibly cause the adverse toxic effect. Thus, the question remains open why the alloy ZK60 processed by multiaxial forging showed higher toxicity than the one processed by the combination of extrusion and ECAP. Perhaps this question can be related to the general challenges of probing the cytotoxicity of magnesium in vitro MTT assays [77]. Nonetheless, the concurrent use of the flow cytometry method strongly corroborates our results drawn from the MTT assay. For the sake of comparison, one can notice, for example, that the viability of the cells in all alloys studied in the present work was notably better than that reported for the popular alloy AZ31 [52] with the grain structure refined by ECAP. Even though the average corrosion rate in SBF of 2.3–3.5 mm/year for most perspective alloys considered in the present study is found to be superior to that of the fine-grained AZ31 [53], there is still a lot to desire in the corrosion resistance, which has to be improved significantly before the alloys can be considered for practical applications in medicine. The time-proven recipe to accomplish this technologically-relevant goal for given chemical compositions is, however, well known and rooted primarily on enhancing the purity of the alloys (particularly with respect to such trace elements as Fe, Ni and Cu) and producing a more uniform microstructure. This will be the scope of future efforts. It is timely to notice that, the experimental conditions in vitro to not perfectly replicate complex physiological conditions and the interactions between the implanted material and tissues in vivo. However, the corrosion rates in vivo are usually lower by a factor of 1-4 than in vitro, exhibiting a correlation between these two rates [78,79] and bring about some optimism in the future of biomedical applications of these materials.

Concluding this comparative study, it is safe to state that contemporary deformation techniques, particularly those united commonly under the term “severe plastic deformation techniques” are capable of manufacturing the low alloyed Mg-based systems with the mechanical properties, which are good enough for a wide variety of medical applications as temporary implants.

Although the microstructure is of key importance in tailoring the mechanical and corrosion (biodegradation) behaviour of magnesium alloys, it is not of decisive importance in the cytotoxic effects. The assessment of in vitro cytotoxicity of several Mg–Zn–X (X = Ca, Zr, Y or none) indicated that all the alloys studied did not induce significant toxicity in the cells and are suitable for biomedical applications with one exception noted above for the specifically manufactured alloy ZK60.

## Figures and Tables

**Table 1:** Chemical compositions of the alloys studied.

N	Alloy (wt.%)		Mg	Zn	Ca	Zr	Y	Al	Fe	Cu	Ni	Si
1	ZK60(I)	Mg-6Zn-0.5Zr	94.52	5.02	0.0003	0.44	0.006	0.0036	0.0032	<0.0001	<0.0001	<0.0001
2	ZK60(II)	Mg-6Zn-0.5Zr	94.3	5.09	0.0003	0.52	0.007	0.0030	0.0036	<0.0001	<0.0001	0.0002
3	Z4	Mg-4Zn	96.18	3.79	0.018	0.002	0.005	0.0096	0.0059	<0.0001	0.0004	<0.0001
4	ZX10	Mg-0.9Zn-0.2Ca	98.90	0.85	0.218	<0.0001	<0.001	0.0085	0.0072	0.0008	0.0014	0.007
5	WZ31	Mg-1Zn-2.8Y	96.40	1.08	0.0006	0.002	2.07	0.005	0.02	<0.0001	0.0057	0.24
6	WZ62	Mg-2.0Zn-5.7Y	92.24	2.0	0.003	0.032	5.72	0.046	0.0004	0.0012	<0.0001	0.007

**Table 2:** Processing conditions and functional properties of Mg alloys.

№	Alloy	Composition (wt. %)	Processing/Temperature	Equivalent Imposed Strain	Grain Size, D/μm	Yield Stress, σ_0.2_/MPa	Ultimate Strength, σ_UTS_/MPa	Elongation at Break ε_f_ (%)	Corrosion Rate in SBF, mm/Year
1	ZK60 (I)	Mg-6Zn-0.5Zr	E + ECAP/350 °C	3.4 + 2.0	7.7 ± 0.5	293 + 5	330 ± 8	16.2 ± 1.7	3.5 ± 0.4
2	ZK60 (II)	Mg-6Zn-0.5Zr	Two-step MIF/400 °С + 300 °С	4.2 + 3.0	5.0 ± 0.3	205 + 7	328 ± 4	30.3 ± 3.3	10.3 ± 0.9
3	Z4	Mg-4Zn	ECAP/415 °C	1.15	126 ± 86	126 + 3	250 ± 2	18.5 ± 2.2	2.3 ± 0.4
4	ZX10	Mg-1Zn-0.1Ca	E/325 °C	1.6	2.1 + 0.4 *	230 + 4	285 ± 3	13.5 ± 2.2	3.2
5	WZ31	Mg-1Zn-2.9Y	ECAP/425 °C	2.30	2.0 ± 1.6	277 + 4	318 ± 3	15.3 ± 3.6	13 ± 2
6	WZ62	Mg-2Zn-5.7Y	E/350 °C	1.6	8.8 ± 4.2	364 + 5	430 ± 2	4.6 ± 2.8	2.3

E—extrusion, ECAP—equal channel angular pressing, MIF—multiaxial isothermal forging * refers to DRX grains.

**Table 3:** Viability (in%) of immortalised human fibroblasts after incubation with extracts of Mg-based alloys for 24 h.

Dilution of the Extract, Times	Specimen *					
	1	2	3	4	5	6
	ZK60 (I)	ZK60 (II)	Z4	ZX10	WZ31	WZ62
0	90.1 ± 10.4	48.1 ± 1.4	96.3 ± 2.6	95.4 ± 7.2	81.4 ± 13.6	100.8 ± 13.0
2	99.0 ± 5.1	84.5 ± 2.7	93.3 ± 8.7	97.6 ± 8.4	87.9 ± 10.1	95.2 ± 2.5
4	107.7 ± 13.1	90.2 ± 6.7	93.3 ± 3.9	99.1 ± 6.2	91.3 ± 1.4	102.0 ± 11.1
6	93.9 ± 1.7	100.8 ± 4.7	93.0 ± 2.3	94.5 ± 3.5	91.6 ± 13.7	96.0 ± 3.6
8	95.7 ± 15.0	95.3 ± 0.8	92.9 ± 1.3	96.8 ± 5.5	97.4 ± 5.0	103.4 ± 9.2
10	96.3 ± 12.0	96.5 ± 4.7	103.2 ± 1.8	97.7 ± 5.4	97.2 ± 15.3	98.3 ± 6.0

* note that for positive control—doxorubicin 5 μM - viability is 33.6 ± 1.6%; for negative control—culture medium without antibiotic - viability is 100%.

**Table 4:** The percentage of necrotic cells in the population of immortalised human fibroblasts after incubation with extracts (iodide propidium staining, analysis of at least 60,000 cells).

Sample		Necrosis of Cells, %	рН of Culture Medium
Control		0.491 ± 0.005	7.77
Mitomycin		1.03 ± 0.01	-
1	ZX60 (I)	3.45 ± 0.03	8.06
2	ZK60 (II)	8.25 ± 0.09	8.25
3	Z4	2.00 ± 0.02	8.11
4	ZX10	1.92 ± 0.02	8.17
5	WZ31	3.63 ± 0.04	8.25
6	WZ62	1.06 ± 0.01	8.15
